# Transcriptional Inhibition of lncRNA gadd7 by CRISPR/dCas9-KRAB Protects Spermatocyte Viability

**DOI:** 10.3389/fmolb.2021.652392

**Published:** 2021-03-11

**Authors:** Jun Zhao, Wenmin Ma, Yucheng Zhong, Hao Deng, Bingyu Zhou, Yaqin Wu, Meiqiong Yang, Huan Li

**Affiliations:** Assisted Reproductive Technology Center, Foshan Maternal and Child Health Care Hospital, Foshan, China

**Keywords:** lncRNA gadd7, CRISPR, varicocele, infertility, male

## Abstract

Our previous study found that lncRNA gadd7 was up-regulated in the semen of varicocele patients, and could promote the apoptosis of mouse spermatocytes and inhibit their proliferation. Therefore, we further explored whether down-regulation of Gadd seven expression could protect the viability of spermatocytes. Here we designed specific sgRNAs targeting the ORF region of gadd7, and constructed a CRISPR-dCas9-KRAB system that effectively inhibits gadd7 at the transcriptional level. The CRISPRi system can effectively prevent the apoptosis of spermatocytes and enhance their proliferation, which is expected to provide a potentially effective molecular intervention method for the treatment of male infertility caused by varicocele.

## Introduction

Varicocele is a common disease of the male reproductive system (Hyun, 2020). The incidence rate in the general male population is 15%, and the incidence rate in the infertile population is as high as 39% (Alsaikhan et al., 2016; Barratt et al., 2017). It is considered by the World Health Organization to be one of the most common causes of male infertility. It affects the spermatogenic function of the testis through a variety of pathophysiological changes. The theory of renal venous reflux is considered to be a possible cause of damage to spermatogenesis (Sayfan and Adam, 1978; Graif et al., 2000). Toxic metabolites from the reflux of kidneys/adrenal glands cause local microenvironmental changes in spermatogenesis of testis. Clinically, semen quality testing is often used to determine the effect of varicocele on men. For patients with moderate to severe varicocele, surgical treatment is the most effective treatment method, and microscopy is the current gold standard for surgical treatment (Johnson and Sandlow, 2017).

Long noncoding RNAs (LncRNAs) are a type of RNA molecules with a transcript length of more than 200 nt (Kopp and Mendell, 2018). They are widely present in mammals and participate in various normal activities of cells, such as genome imprinting, transcription regulation, X Chromosome silencing and nuclear transport (Chen, 2016). They also participate in the pathological process of tumors and other diseases. LncRNAs do not encode proteins, but they regulate gene expression at multiple levels in the form of RNA, and are related to a series of major human diseases, especially neurological diseases and cancers (Renganathan and Felley-Bosco, 2017; Choudhari et al., 2020).

In recent years, the research on LncRNAs has progressed rapidly, but the function and mechanism of most LncRNAs are still unclear. The gene expressing gadd7 was cloned from Chinese hamster ovary cells exposed to ultraviolet light in 1988 (Hollander et al., 1996). It is a LncRNA specifically expressed in hamster cells. Basically all factors such as growth arrest, DNA damage, and excessive oxidative stress can induce its expression (Mizenina et al., 1998). Currently, there still lacks much research on the function and mechanism of its action. Studies have found that, in addition to inhibiting cell clone formation, the lncRNA gadd7 also plays an important regulatory role in lipotoxicity-induced cell death. Our previous studies have found that gadd7 is elevated in the sperm of patients with varicocele, and can promote the apoptosis of mouse spermatocytes and inhibit their proliferation (Zhao et al., 2018).

A more interesting idea is whether down-regulation of lncRNA gadd7 can protect spermatocytes. However, traditional RNAi technology is difficult to achieve effective knockdown of lncRNA, which limits the implementation of this idea. The CRISPRi transcription suppression technology developed in recent years is expected to inhibit the expression of lncRNA (Liu et al., 2017; Stojic et al., 2018; Liu et al., 2020). The dCas9 protein targeted to the ORF region of the gene can silence the expression of specific lncRNA by fusing the KRAB transcription repressor. In this study, we designed a specific sgRNA for the ORF region of gadd7, and screened and obtained a CRISPRi system that effectively inhibits gadd7. This system can effectively slow down the apoptosis of spermatocytes and promote their proliferation, which is expected to provide a potentially effective molecular intervention method for the treatment of male infertility caused by varicocele.

## Materials and Methods

### Cell Lines and Cell Culture

The two mouse germ cell lines, GC-1 and GC-2, had been purchased from the Institute of Cell Research, Chinese Academy of Sciences (Shanghai, China). Cells were grown in RPMI 1640 medium supplemented with 10% fetal bovine serum (Invitrogen) at 37°C in a 5% CO_2_ atmosphere.

### Construction of CRISPR-dCas9-KRAB and Transfection

The plasmid vectors pcDNA-dCas9-KRAB (#110820, Addgene) was used to transiently express dCas9-KRAB fusion protein (for CRISPR-based interference) in mouse germ cells. The designed cDNA sequence for each sgRNA was designed using the online software tool (http://crispr-era.stanford.edu/), and synthesized and inserted into pGPU6/GFP/Neo vector which was digested with Bam HI/Bbs I. The leader sequence of sgRNA negative control was 5′-GTA​CGT​TCT​CTA​TCA​CTG​ATA-3′.

The above plasmids were transfected into GC-1 and GC-2 cells using Nanofectin™ Transfection reagent (Excell Bio, Shanghai, China) according to the supplier’s protocol. The final concentration of plasmid was 1 μg/ml.

### Quantitative Real-Time PCR (qRT-PCR)

Total RNA was extracted from indicated cells or frozen specimens using Trizol reagents (Invitrogen). Reverse transcription was performed using M-MLV Reverse transcriptase (Invitrogen) and the extracted RNA. qRT-PCR was conducted on the Applied Biosystems 7,300 Fast Real-Time PCR System (Applied Biosystems, Foster City, CA, United States) using SYBR^®^ Green Realtime PCR Master Mix (Toyobo, Osaka, Japan) and the primers 5′-ACA​ATG​ACG​CCA​TCG​TTT​TCT-3' (forward) and 5′- TGT​CCT​CCA​TCT​GGG​CAT​TT-3-3' (reverse) for gadd7. The expression levels were calculated using the comparative CT method for relative quantification against GAPDH. The primers for GAPDH were forward 5′-CGC​TCT​CTG​CTC​CTC​CTG​TTC-3′, and reverse 5′-ATC​CGT​TGA​CTC​CGA​CCT​TCA​C-3′.

### Cell Proliferation Assay

Cell proliferation abilities were evaluated using Cell counting kit-8 (CCK-8).Briefly, 3,000 indicated GC-1/GC-2 cells were plated into 96-well plates per well. After incubation for 24 h, 48 h, or 72 h, 10 μL CCK-8 solution (Biyuntian Biological Engineering Co. Ltd., Shanghai, China) was added to per well. After incubation for 2 h, the absorbance at 450 nm was measured at 490 nm by a microplate reader (Bio-Rad, Hercules, CA). Each experiment was done at least three times.

### Cell Apoptosis Assays

GC-1 and GC-2 cells were transiently transfected with plasmid vectors, and then cells were harvested and resuspended in fixation fluid 48 h after transfection. 5 μl of Annexin V—FIFC and 2 μl of propidium iodide were added to 500 μl of cell suspension. Cell apoptosis was then determined using flow cytometry (EPICS, XL- 4, Beckman, CA, United States). Each experiment was done at least three times.

The caspase-3 activity in GC-1 and GC-2 cells was determined by ELISA using a Caspase-3 Activity Assay kit (Fluorometric). At 48 h after transfection, cells were reaped and cultured in lysis buffer for 15 min at 4°C, followed by centrifugation and supernatant collection. The final absorbance (at 405 nm) was determined using a microplate reader.

### Statistical Methods

The *t* test (homogeneity of variance) or t 'test (heterogeneity of variance) of two or more independent samples was used for the normal distribution data. SPSS 17.0 software was used for analysis, and *p* < 0.05 was considered statistically significant.

## Results

### Design and Construction of CRISPR-dCas9-KRAB Targeting gadd7

**FIGURE 1:**
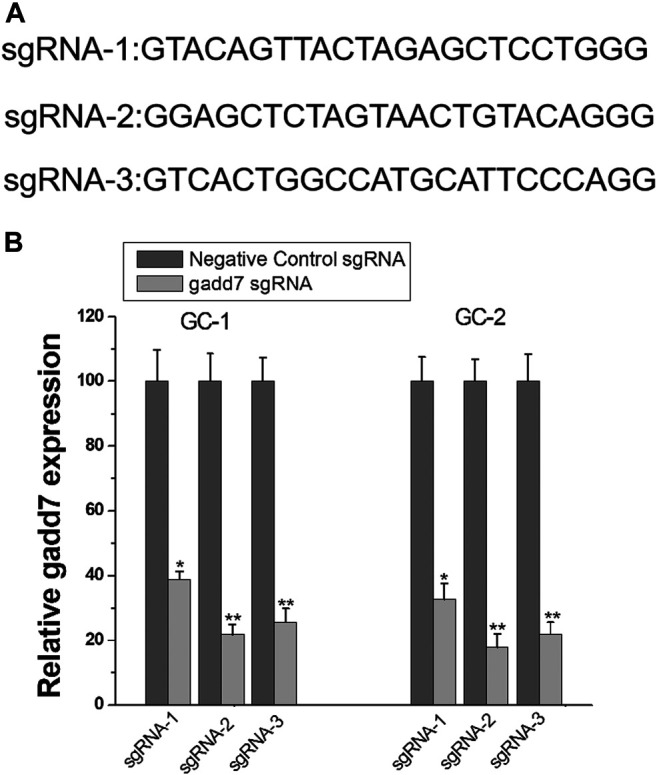
CRISPR-dCas9-KRAB inhibits lncRNA gadd7 expression. **(A)** cDNA sequences of the first 20 nt of the sgRNAs targeting the ORF region of gadd7. **(B)** Inhibition of gadd7 expression using the designed sgRNAs in GC-1 and GC-2 cells. The relative expression level of gadd7 wad determined by qRT-PCR. *p < 0.05, relative to the negative control by paired, one-sided *t*-test. **p < 0.01, relative to the negative control by paired, one-sided *t*-test.

To determine whether the CRISPRi technology can be used to inhibit the expression of lncRNA gadd7, we targeted the dCas9-KRAB protein to the ORF regions of gadd7 using designed sgRNAs (sgRNA1∼3) (Figure 1A). Then, we introduced dCas9-KRAB and sgRNA combination into GC-1 and GC-2 cells. The expression of these sgRNAs induced various significant decreases in gadd7 expression, and the strongest inhibition was achieved with sgRNA-2 (Figure 1B). Therefore, we chose sgRNA-2 to do the following experiments.

### Downregulation of gadd7 by CRISPR-dCas9-KRAB Reduced Cell Apoptosis

**FIGURE 2:**
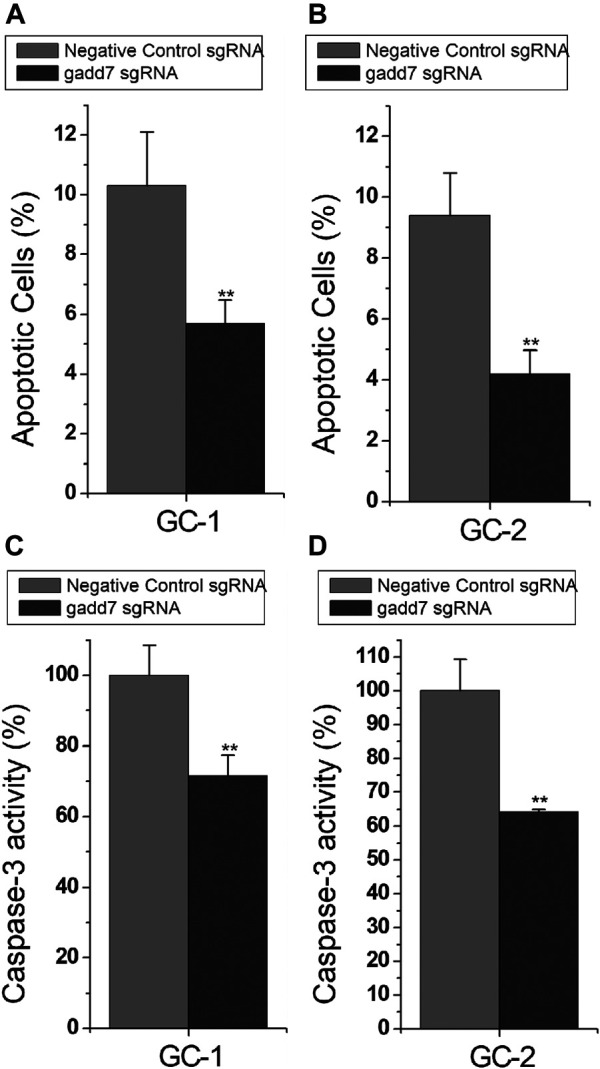
Inactivation of gadd7 by CRISPR-dCas9-KRAB prevents cell apoptosis. **(A)** Cell apoptosis changes were determined using flow cytometry analysis in GC-1 cells. **(B)** Cell apoptosis changes in GC-2 cells. **(C)** Caspase-3 activity was determined using ELISA in GC-1 cells. **(D)** Caspase-3 activity was determined using ELISA in GC-2 cells. The error bars indicate the S.D. from three different experiments. NC, negative control. **p < 0.01, relative to the negative control group by paired, one-sided *t*-test.

To investigate the downstream effect of downregulation of gadd7 on the apoptosis of GC-1 and GC-2 cells, the cell apoptotic rates of these cells were determined using the Flow cytometry assay. The results shown in Figure 2 demonstrated that the apoptotic cells (%) of GC-1 (Figure 2A) and GC-2 (Figure 2B) cell lines transfected with the CRISPR-dCas9-KRAB system were much lower than those transfected with the negative control plasmid. These results suggested that downregulation of gadd7 by CRISPR-dCas9-KRAB could prevent the apoptosis of germ cells.

### Downregulation of gadd7 by CRISPR-dCas9-KRAB Enhanced Cell Proliferation

**FIGURE 3:**
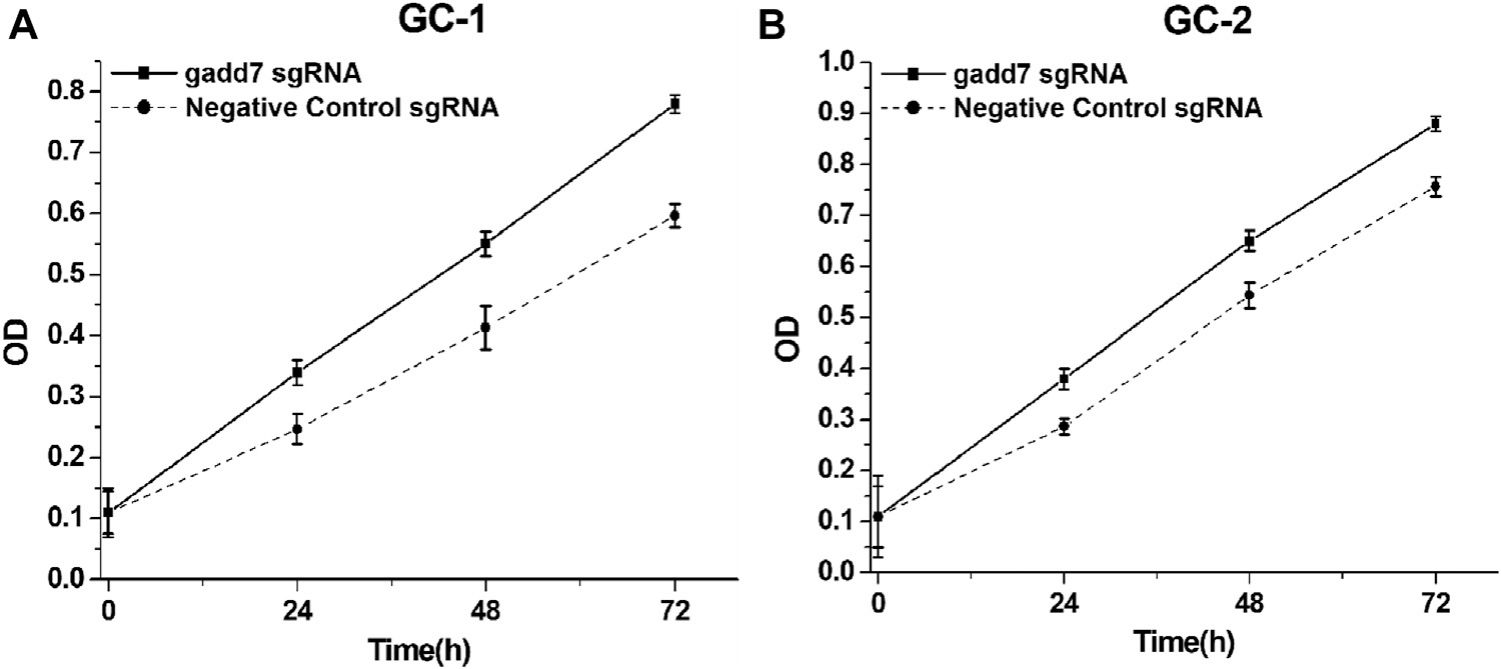
Inactivation of gadd7 by CRISPR-dCas9-KRAB enhances cell proliferation. Cell proliferation was detected by CCK-8 assay. **(A)** Compared to the negative control group, the proliferative ability of GC-1 cells transfected with gadd7 sgRNA was significantly increased (*p* < 0.01). **(B)** Compared to the negative control group, the proliferative ability of GC-2 cells transfected with gadd7 sgRNA was significantly increased (*p* < 0.01).

To investigate the downstream effect of downregulation of gadd7 on the proliferation of GC-1 and GC-2 cells, the cell proliferation rates of these cells were determined using the CCK-8 assay. The results shown in Figure 2 demonstrated that the proliferation rate of GC-1 (Figure 3A) and GC-2 (Figure 3B) cell lines transfected with the CRISPR-dCas9-KRAB system were much higher than those transfected with the negative control plasmid. These results suggested that downregulation of gadd7 by CRISPR-dCas9-KRAB could enhance the proliferation of germ cells.

## Discussion

It has always been the dream of many biologists to have a technology that can control gene expression at will. The emergence of CRISPR/Cas9 system meets this need. Cas9 is like a DNA scissors. Under the guidance of sgRNA, it specifically cuts the target sequence and forms DNA double strand breaks (Jiang and Doudna, 2017; Kosicki et al., 2018). Later studies obtained dead Cas9 (dCas9) by inactivating the endonuclease activity of Cas9. dCas9 only binds to the target site under the guidance of sgRNA, but does not cleave DNA. The coupling of dCas9 with epigenetic modifiers can efficiently regulate the transcription of specific genes (Komor et al., 2016; Savić and Schwank, 2016). When the kox1 domain of KRAB is fused with dCas9, the CRISPR system can be used to induce transcriptional suppression. In addition to the regulation of protein coding region, CRISPR-dCas9 system can also act on non coding RNAs including lncRNAs.

In this study, we designed a CRISPR-dCas9-KRAB transcriptional regulation system which inhibits mouse lncRNA gadd7. To the best of our knowledge, this is the first report which used CRISPR-dCas9 to regulate gene expression in spermatocytes. By inhibiting the expression of gadd7, this system can protect the viability of spermatocytes, which is manifested in the reduction of apoptosis and enhancement of proliferation. These results suggest that CRISPRi technique may be used in male oligoasthenospermia caused by varicocele. There may be other lncRNAs with functions similar to gadd7 that can affect spermatocytes, which need to be further explored.

In the future, further animal experiments are needed to verify the biological protection effect of the system *in vivo*.

## Data Availability

The original contributions presented in the study are included in the article/Supplementary Material, further inquiries can be directed to the corresponding authors.
